# Predictive value of GGN and CAG repeat polymorphisms of androgen receptors in testicular cancer: a meta-analysis

**DOI:** 10.18632/oncotarget.7337

**Published:** 2016-02-12

**Authors:** Weijun Jiang, Jing Zhang, Qing Zhou, Shuaimei Liu, Mengxia Ni, Peiran Zhu, Qiuyue Wu, Weiwei Li, Mingchao Zhang, Xinyi Xia

**Affiliations:** ^1^ Department of Reproduction and Genetics, Institute of Laboratory Medicine, Jinling Hospital, Nanjing University School of Medicine, Nanjing 210002, P.R. China

**Keywords:** androgen receptor, GGN repeat, CAG repeat, testicular cancer, polymorphism

## Abstract

The risk of testicular cancer (TC) is markedly increased in subjects with androgen insensitivity, and previous studies have proposed that GGN and CAG repeats in androgen receptors (*AR*) could be related to the risk of TC. To evaluate the association between the length of GGN and CAG repeats in *AR* and TC, a meta-analysis involving 3255 TC cases and 2804 controls was performed. The results suggested that long GGN repeats are associated with an increased risk of TC compared with those < 23 [odds ratio (OR) = 1.22, 95% confidence interval (CI) = 1.05–1.41]; similarly, a subgroup analysis revealed that this association occurred in studies with case sizes > 200, and in the mid-latitude, and seminoma subgroups. The subgroup analysis based on populations, high-latitude, and seminomas/non-seminomas suggested that AR CAG repeat polymorphisms with > 25 and < 21 + > 25 repeats might confer a protective effect to the patients with TC (in the high-latitude subgroup analysis, for > 25 *vs*. 21–25: OR = 0.54, 95% CI = 0.41–0.70). In contrast, an increased risk of TC was observed for *AR* CAG repeat polymorphisms with > 25 and < 21 + > 25 repeats in the mid-latitude subgroup (for > 25 *vs*. 21–25: OR = 1.65, 95% CI = 1.09–2.50). In addition, no associations between the remaining subgroups and male infertility were observed. In short, this meta-analysis suggested that *AR* GGN and CAG repeat polymorphisms may be involved in the etiology of TC.

## INTRODUCTION

Testicular cancer (TC) is an malignancy, accounting for 1%–2% of all tumors among men worldwide [1], and affects primarily young men in the age group 15–44 years. The incidence of TC is increasing worldwide and has steeply increased in the past 40 years in almost all Western countries [2–4]. Clinical studies reported that 95% of all TCs are testicular germ cell tumors (TGCT), with an approximately equal division between seminomas and non-seminomas, and epidemiological studies have suggested that environmental factors, including endocrine disrupting agents, which act as either weak estrogen agonists or androgen antagonists, are primarily responsible for the increased incidence of TC [5, 6].

The *AR* gene, located on Xq11-12, has 8 exons and 7 introns, and in exon 1, this gene contains two important polymorphic trinucleotide repeats of polyglutamine and polyglycine tracts [7, 8], which are encoded by a (CAG) _n_CAA stretch, as well as a (GGT)_3_GGG(GGT)_2_(GGC)_n_ repeat, and these repeats are designated CAG and GGN repeats, respectively [9]. The extreme variability of the number of these repeats determines the different lengths of the polyglutamine and polyglycine segments in the N-terminal transactivation domain of the *AR* [10]. In men, the number of CAG repeats can vary from 8 to 37, with an average of 20–22, depending on the ethnic origin. Africans and Asians have a lower number of repeats than Caucasians and a reduced risk of TGCT [10]. Changes in the length of the CAG polymorphic trinucleotide repeat in the *AR* gene may lead to the altered transactivation of the *AR* gene and have been implicated to play a role in the pathogenesis of several forms of endocrine cancer and certain reproductive disorders [11]. Subjects with reproductive disorders that are associated with a relative deficiency in androgen function have an increased risk of TC [12, 13]. In the past decade, some studies have attempted to evaluate the association between CAG and GGN repeat number and the risk of TC [6, 9, 10, 12–15]; however, the results appear contradictory because of differences in the sources of the study participants and inconsistencies in the inclusion criteria in case and control subjects among the studies [9, 16]. To the best of our knowledge, to date, no meta-analysis has analyzed the results of all the studies that evaluated this association. Therefore, this meta-analysis was conducted to investigate the association between CAG and GGN repeat polymorphisms and the risk of TC, as well as the genetic heterogeneity across different control sources and study designs. Herein, seven reports involving 3255 TC cases and 2804 controls were identified according to the inclusion criteria for the pooled analysis.

## RESULTS

### Study characteristics

**Figure 1:**
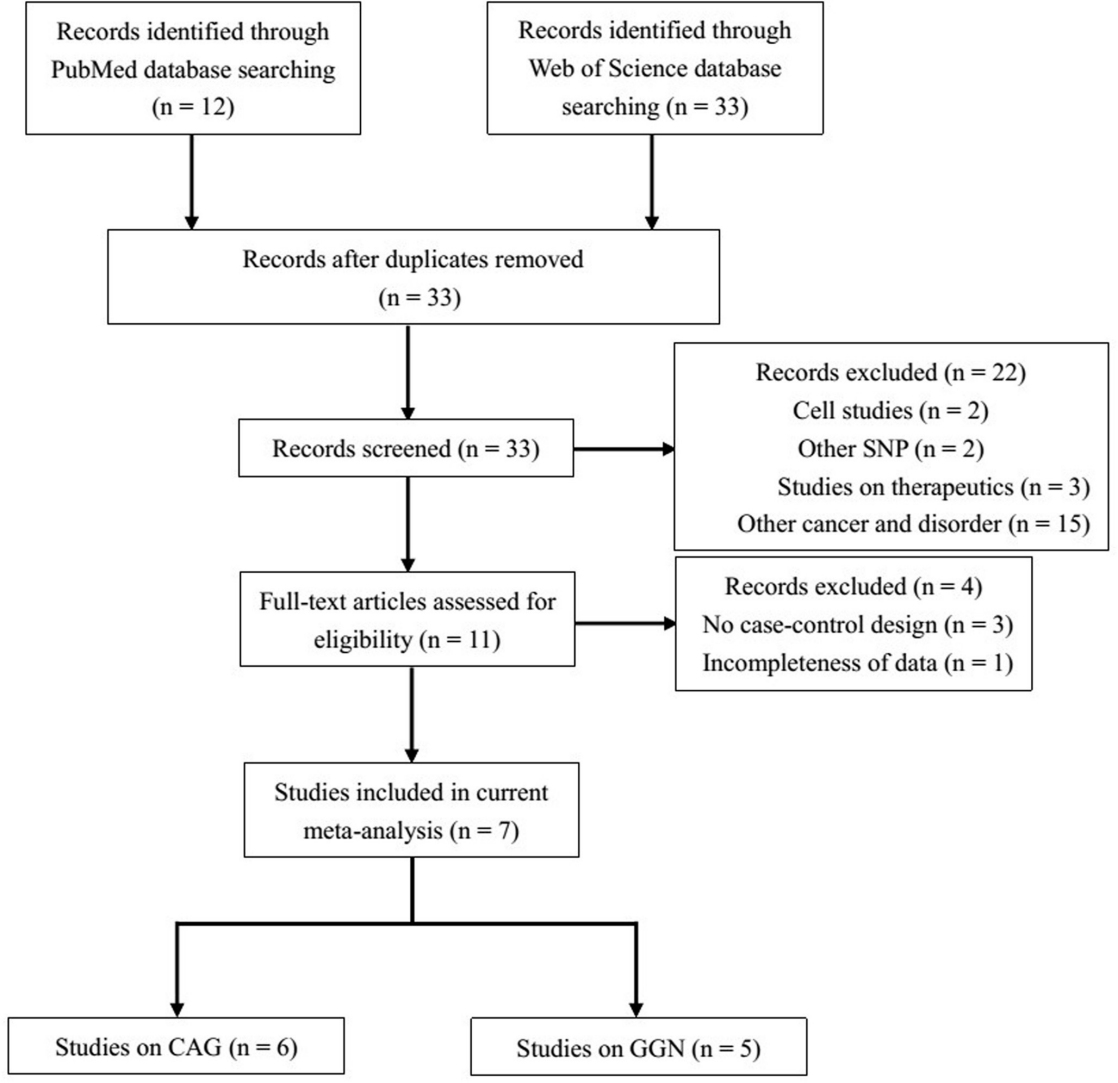
Flow diagram of the study selection process

Because not all of the studies evaluated provided specific distributions of *AR* CAG or GGN repeat counts, we used a CAG repeat length of 21–25 as reference to evaluate dichotomous comparisons (< 21 CAG repeats *vs.* the reference, > 25 CAG repeats *vs.* the reference, and < 21 + > 25 CAG repeats *vs.* the reference). Similarly, the GGN genotype of ≤ 23 repeats was used as reference to assess the association between > 23 repeats and the risk of TC. Through literature search and selection based on the inclusion criteria, 7 articles published between 2002 and 2015 were identified after reviewing potentially relevant articles (Figure 1). The characteristics of the enrolled studies are summarized in Tables 1 and 2.

**Table 1:** Main characteristics of the studies on the GGN repeats included in the meta-analysis

Author (year)	Country	Ethnicity	Method	Control source	Latitude	Case size	Cases/ Controls	Group	Case		Control	
									≤ 23	> 23	≤ 23	> 23
Grassetti D (2015)	Italy	Caucasian	Sequence	HB	Mid-latitude	> 200	302/322	Total	182	120	206	116
							166	Seminomas	97	69		
							136	Non-seminomas	85	51		
Kristiansen W (2012)	Norway	Caucasian	Sequence	PB	High-latitude	> 200	635/292	Total	391	244	196	96
							315	Seminomas	191	124		
							320	Non-seminomas	200	120		
Vastermark A (2011a)	Sweden	Caucasian	Sequence	PB	High-latitude	> 200	267/214	Total	175	92	141	73
Vastermark A (2011b)	Denmark	Caucasian	Sequence	PB	High-latitude	< 200	74/214	Total	43	31	141	73
							158	Seminomas	101	57		
							178	Non-seminomas	114	64		
Biggs ML (2008)	USA	Mixed	Sequence	PB	Mid-latitude	> 200	235/576	Total	133	102	359	217
Garolla A (2005)	Italy	Caucasian	Sequence	PB	Mid-latitude	< 200	123/115	Total	69	54	71	44

Abbreviations: HB, hospital-based; PB, population-based.

**Table 2:** Main characteristics of the studies on the CAG repeats included in the meta-analysis

Author (year)	Country	Ethnicity	Method	Control source	Latitude	Case size	Cases/controls	Group	Case			Control		
									< 21	21–25	> 25	< 21	21–25	> 25
Grassetti D (2015)	Italy	Caucasian	Sequence	HB	Mid-latitude	> 200	302/322	Total	83	171	48	74	211	37
Kristiansen W (2012)	Norway	Caucasian	Sequence	PB	High-latitude	> 200	635/304	Total	189	374	72	91	174	72
							316	Seminomas	92	187	37			
							319	Non-seminomas	97	187	35			
Vastermark A (2011a)	Sweden	Caucasian	Sequence	PB	High-latitude	> 200	275/214	Total	89	161	25	72	112	30
Vastermark A (2011b)	Denmark	Caucasian	Sequence	PB	High-latitude	< 200	89/214	Total	31	52	6	72	112	30
							172	Seminomas	59	97	16			
							186	Non-seminomas	60	111	15			
Garolla A (2005)	Italy	Caucasian	Sequence	PB	Mid-latitude	< 200	123/115	Total	38	67	18	37	68	10
Giwercman A (2004)	Sweden	Caucasian	Sequence	PB	High-latitude	< 200	83/220	Total	28	41	14	78	99	43
							27	Seminomas	8	19	0			
							41	Non-seminomas	15	18	8			
Rajpert-De Meyts E (2002)	Denmark	Caucasian	Sequence	HB	High-latitude	< 200	102/110	Total	32	61	9	43	55	12

Abbreviations: HB, hospital-based; PB, population-based.

For the GGN repeats, seven studies involving 1636 cases (range of 74–635, average of 272.67 ± 180.5) and 1519 controls (range of 115–576, average of 304 ± 154) were included in the meta-analysis.

For the CAG repeats, 6 studies involving 1609 cases (range of 83–635, average of 230 ± 185) and 1285 controls (range of 110–322, average of 214 ± 82.1) met the inclusion criteria and were selected for the meta-analysis.

### Association between GGN repeat length and the risk of TC

**Figure 2:**
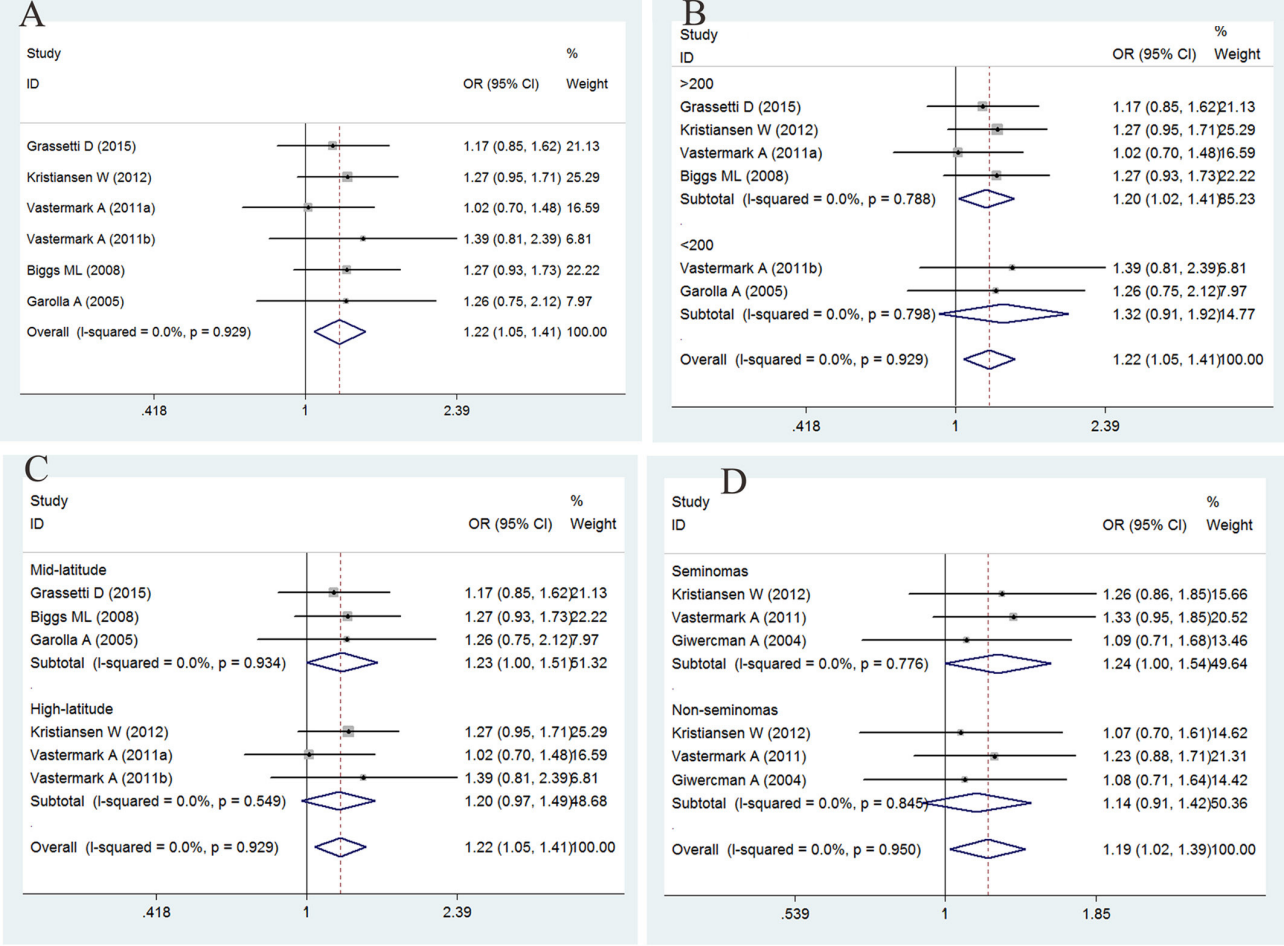
Forest plot of the GGN repeat polymorphism and the risk of testicular cancer (**A**) Overall analysis. (**B**) Case size subgroup. (**C**) Latitude subgroup. (**D**) Histology subgroup. Studies are plotted according to the last name of the first author, followed by the publication year in parentheses. Each square represents the OR point estimate and its size is proportional to the weight of the study. The diamond (and broken line) represents the overall summary estimate and its width indicates the confidence interval. The unbroken vertical line is at the null value (OR = 1.0).

The association between GGN repeats and the risk of TC is summarized in Figure 2 and Table 3. The overall analysis indicated a significant association between GGN repeats and TC [odds ratio (OR) = 1.22, 95% confidence interval (CI) = 1.05–1.41, *P* = 0.010]. To clarify the potential effect of latitude, sample size, and histological differences, a subgroup analysis of study populations was also conducted, and a significant association was found between GGN repeats and TC in studies with a sample size > 200 and in the mid-latitude and seminoma subgroups (for > 23 *vs.* ≤ 23: OR = 1.23, 95% CI = 1.00–1.51, *P* = 0.050; for > 23 *vs.* ≤ 23: OR = 1.20, 95% CI = 1.02–1.41, *P* = 0.028; for > 23 *vs.* ≤ 23: OR = 1.24, 95% CI = 1.00–1.54, *P* = 0.050).

**Table 3:** Main results for the GGN repeats included in the meta-analysis

	Cases/Controls	OR (95% CI)	*P*	*P*_h_	*I*^2^	*Z*	*P*_b_
Overall	1636/1519	**1.22 (1.05–1.41)**	**0.010**	0.929	0.0%	2.59	0.840
**Case size**							
> 200	1439/1190	**1.20 (1.02–1.41)**	**0.028**	0.788	0.0%	2.19	
< 200	197/329	1.32 (0.91–1.92)	0.143	0.798	0.0%	1.46	
**Latitude**							
Mid-latitude	660/1013	**1.23 (1.00–1.51)**	**0.050**	0.934	0.0%	1.96	
High-latitude	976/506	1.20 (0.97–1.49)	0.089	0.549	0.0%	1.7	
**Histology**							
Seminomas	639/828	**1.24 (1.00–1.54)**	**0.050**	0.776	0.0%	1.96	
Non-seminomas	634/828	1.14 (0.91–1.42)	0.249	0.845	0.0%	1.15	

Note: *P*_h_ value of the *Q*-test for the heterogeneity test; *P*_b_ value of Egger's test for publication bias.

*I*^2^: 0–25, absence of heterogeneity; 25–50, modest heterogeneity; 50, high heterogeneity.

Bold numbers indicate significant differences.

### Association between CAG repeat length and the risk of TC

**Figure 3:**
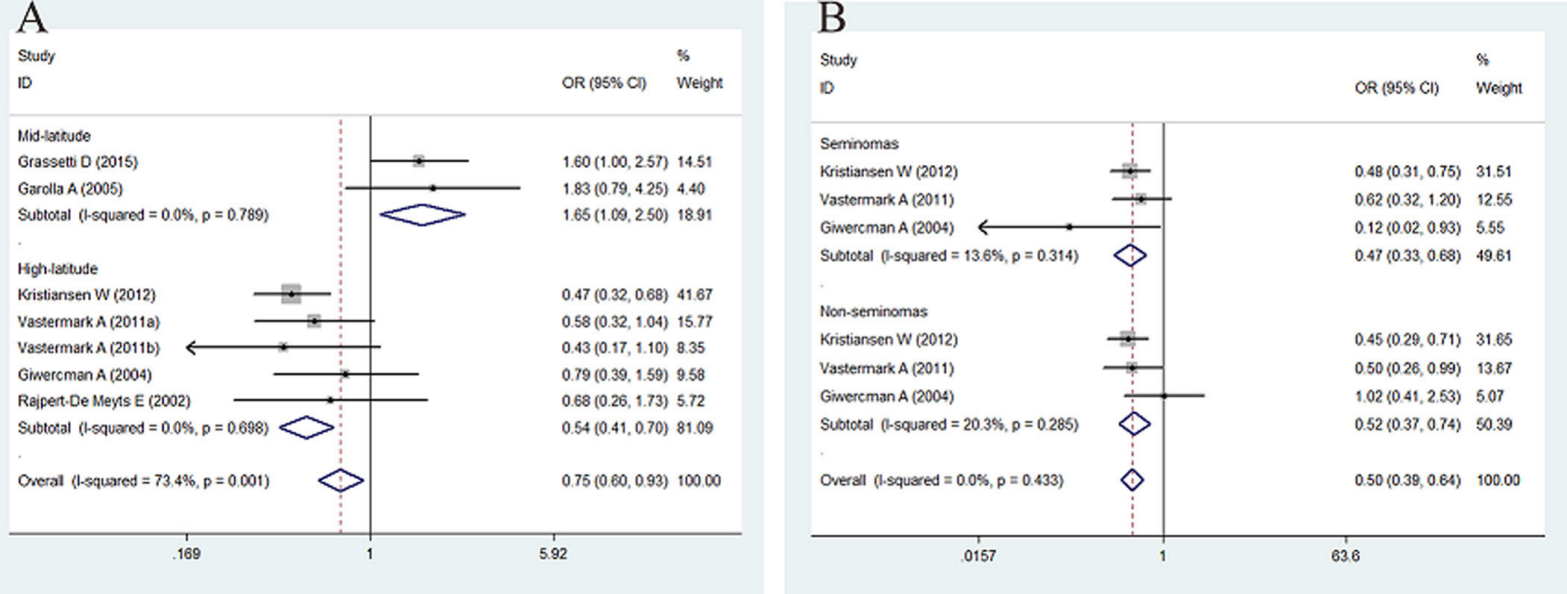
Forest plot of the long CAG repeat polymorphism and the risk of TC in the subgroup analysis (**A**) Latitude subgroup. (**B**) Histology subgroup. Studies are plotted according to the last name of the first author, followed by the publication year in parentheses. Each square represents the OR point estimate and its size is proportional to the weight of the study. The diamond (and broken line) represents the overall summary estimate and its width indicates the confidence interval. The unbroken vertical line is at the null value (OR = 1.0).

**Figure 4:**
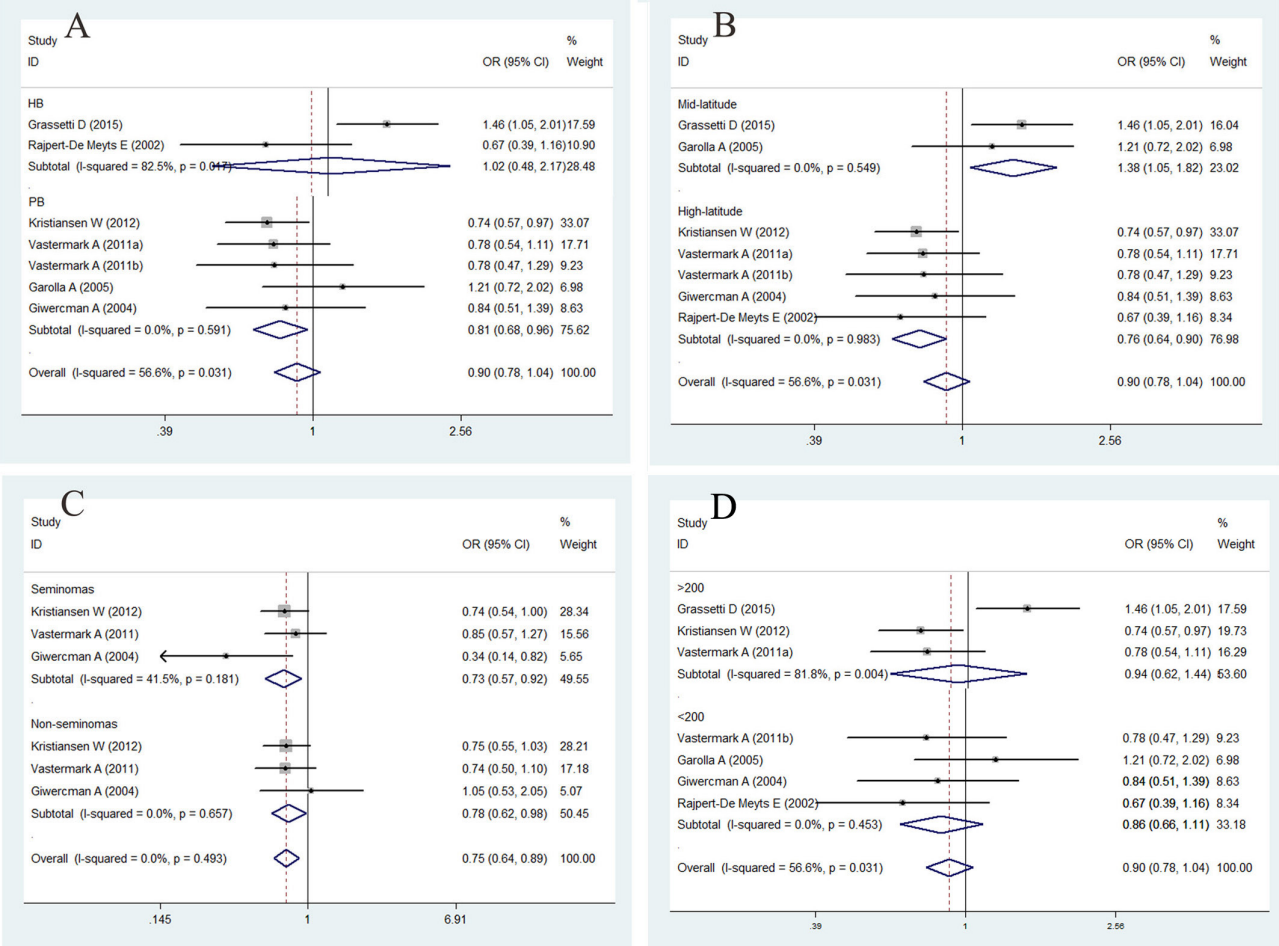
Forest plot of the abnormal CAG repeat polymorphism and the risk of testicular cancer in the subgroup analysis (**A**). Control source subgroup. (**B**). Latitude subgroup. (**C**). Histology subgroup. (**D**). Case size subgroup. Studies are plotted according to the last name of the first author, followed by the publication year in parentheses. Each square represents the OR point estimate and its size is proportional to the weight of the study. The diamond (and broken line) represents the overall summary estimate and its width indicates the confidence interval. The unbroken vertical line is at the null value (OR = 1.0).

The association between CAG repeat in *AR* and the risk of TC is summarized in Figures 3 and 4 and in Table 4. In brief, the overall analysis indicated no significant association between CAG repeats and the risk of TC for the models evaluated; however, in the subgroup analysis based on latitude, sample size, control source, and histology, significant associations were found between CAG repeats and TC in the population-based (PB) subgroup (for < 21 + > 25 *vs.* 21–25: OR = 0.81, 95% CI = 0.68–0.96, *P* = 0.017), mid-latitude subgroup (for > 25 *vs.* 21–25: OR = 1.65, 95% CI = 1.09–2.50, *P* = 0.017; for < 21 + > 25 *vs.* 21–25: OR = 1.38, 95% CI = 1.05–1.82, *P* = 0.021), high-latitude subgroup (for > 25 *vs.* 21–25: OR = 0.54, 95% CI = 0.41–0.70, *P* = 0.000; for < 21 + > 25 *vs.* 21–25: OR = 0.76, 95% CI= 0.64–0.90, *P* = 0.002), seminoma subgroup (for > 25 *vs.* 21–25: OR = 0.47, 95% CI = 0.33–0.68, *P* = 0.000; for < 21 + > 25 *vs.* 21–25: OR = 0.73, 95% CI = 0.57–0.92, *P* = 0.008), and non-seminoma subgroup (for > 25 *vs.* 21–25: OR = 0.52, 95% CI = 0.37–0.74, *P* = 0.000; for < 21 + > 25 *vs.* 21–25: OR = 0.78, 95% CI = 0.62–0.98, *P* = 0.032).

**Table 4:** Main results for the CAG repeats included in the meta-analysis

Cases/Controls		< 21 *vs*. 21–25						> 25 *vs*. 21–25						< 21 + > 25 *vs*. 21–25					
		OR (95% CI)	*P*	*P_h_*	*I^2^*	*Z*	*P_b_*	OR (95% CI)	*P*	*P_h_*	*I^2^*	*Z*	*P_b_*	OR (95% CI)	*P*	*P_h_*	*I^2^*	*Z*	*P_b_*
Overall	1609/1285	0.98 (0.83–1.15)	0.809	0.469	0.0%	0.24	0.371	0.78 (0.49–1.25)	0.304	0.001	73.4%	1.03	0.671	0.90 (0.71–1.14)	0.381	0.031	56.6%	0.88	0.941
**Control source**																			
HB	404/432	1.00 (0.49–2.02)	0.999	0.041	76.1%	0.00		1.15 (0.50–2.61)	0.741	0.108	61.3%	0.33		1.02 (0.48–2.17)	0.955	0.017	82.5%	0.06	
PB	1205/853	0.93 (0.77–1.13)	0.467	0.980	0.0%	0.73		0.66 (0.42–1.04)	0.071	0.049	58.1%	1.81		**0.81 (0.68–0.96)**	0.017	0.591	0.0%	2.39	
**Case size**																			
> 200	1212/626	1.04 (0.85–1.28)	0.687	0.185	40.7%	0.40		0.75 (0.34–1.65)	0.481	0.000	88.0%	0.71		0.94 (0.62–1.44)	0.786	0.004	81.8%	0.27	
< 200	397/659	0.87 (0.66–1.15)	0.335	0.752	0.0%	0.96		0.82 (0.55–1.23)	0.340	0.141	45.0%	0.95		0.86 (0.66–1.11)	0.244	0.453	0.0%	1.16	
**Latitude**																			
Mid-latitude	425/437	1.27 (0.93–1.73)	0.132	0.411	0.0%	1.51		**1.65 (1.09–2.50)**	0.017	0.789	0.0%	2.39		**1.38 (1.05–1.82)**	0.021	0.549	0.0%	2.31	
High-latitude	1085/848	0.89 (0.73–1.08)	0.220	0.873	0.0%	1.23		**0.54 (0.41–0.70)**	0.000	0.698	0.0%	4.62		**0.76 (0.64–0.90)**	0.002	0.983	0.0%	3.11	
**Histology**																			
Seminomas	515/738	0.89 (0.69–1.16)	0.396	0.481	0.0%	0.85	0.182	**0.47 (0.33–0.68)**	0.000	0.314	13.6%	4.06		**0.73 (0.57–0.92)**	0.008	0.181	41.5%	2.66	
Non-seminomas	546/738	0.94 (0.73–1.22)	0.652	0.802	0.0%	0.45	0.870	**0.52 (0.37–0.74)**	0.000	0.285	20.3%	3.65		**0.78 (0.62–0.98)**	0.032	0.657	0.0%	2.14	

Abbreviations: HB, hospital-based; PB, population-based.

Note: *P*_h_ value of *Q*-test for heterogeneity test; *P_b_* value of egger's test for publication bias.

*I*^2^: 0–25, absence of heterogeneity; 25–50, modest heterogeneity; 50, high heterogeneity.

Bold numbers indicate significant differences.

### Publication bias and small-study effects

To assess the publication bias of the studies, Begg's funnel plot and Egger's test were performed. The shapes of the funnel plots revealed no evidence of obvious asymmetry. Egger's test was used to provide statistical evidence of funnel plot symmetry (Supplementary Figure S1) and indicated no evidence of publication bias or small-study effects across the studies (for GGN repeats > 23 *vs.* ≤ 23, *P* = 0.840; for CAG repeat < 21 *vs.* 21–25, *P* = 0.371; for CAG repeats > 25 *vs.* 21–25, *P* = 0.671; for CAG repeats < 21 + > 25 *vs.* 21–25, *P* = 0.941).

### Sensitivity analysis

To confirm the results of the current study, the *I*^2^ statistics calculated for the overall analysis of the CAG repeats > 25 *vs.* 21–25 and CAG repeats < 21 + > 25 *vs.* 21–25 were 73.4% and 56.6%, respectively, indicating that more than 50% of the abnormal CAG repeats may be due to between-study heterogeneity. We then evaluated the source of heterogeneity in these comparisons by sample size, latitude, and histology stratifications, and we observed no heterogeneity in latitude and histology stratifications (Figure 4). Sensitivity analyses were conducted to determine whether modifications in the inclusion criteria of the meta-analysis affected the results. Our results indicated that the studies by Grassetti *et al.* [10] and Garolla *et al.* [17] caused this heterogeneity.

## DISCUSSION

The present meta-analysis, including 3255 TC cases and 2804 controls from seven case-control studies, explored the association between GGN and CAG repeat polymorphisms in *AR* and the risk of TC. Our results indicated that long GGN repeats were associated with an increased risk of TC, compared with repeats < 23; similarly, sample size > 200 and the mid-latitude and seminoma subgroups were associated with an increased risk of TC. In contrast, *AR* CAG repeat polymorphism with > 25 and < 21 + > 25 repeats may confer a protective effect to the TC patients in the analysis of the PB, high-latitude, seminoma, and non-seminoma subgroups. However, *AR* CAG repeat polymorphism with > 25 and < 21 + > 25 repeats in the mid-latitude subgroup were associated with an increased risk of TC.

TC is a very common disease and its incidence has increased worldwide in recent decades. TGCT makes up 95% of all TCs and is the most common solid tumor in men aged 15–39 years [2, 10]. Although there has been enormous progress in the clinical treatment of TC and preservation of fertility through sperm banks in recent years, the main causes of the disease remain unclear. However, important risk factors include work, lifestyle, diet, familial history, environmental conditions, and genetic susceptibility [18–20]. The development of TC is postulated to be due to endocrine disruption, particularly abnormalities in the action of gonadotropins and steroidal sex hormones [17]. Men with androgen insensitivity syndrome caused by *AR* gene mutations have a higher risk of developing TC. There is evidence of an inverse correlation between the variability in *AR* CAG and GGN repeat numbers and the transactivation efficiency in *AR* [6, 9, 10, 15]. Irvine *et al.* [21] suggested that a longer CAG and GGN repeat region might reduce the transactivation activity in *AR*.

Abnormalities in *AR* genes are also common in other disorders, such as prostate cancer, hypospadias, cryptorchidism, and infertility [22–25]. Many authors have attempted to understand whether reduced androgen sensitivity is caused by point mutations or by excessively long CAG and GGN repeat segments, which might lead to the development of testicular agenesis and consequently increase susceptibility to TC [10, 26].

Giwercman *et al.* [13] and Rajpert-De Meyts *et al.* [12] investigated the correlation between CAG and GGN repeats and TC. No statistically significant differences in CAG or GGN repeat numbers were observed between patients with TGCT and the control group. This was the first study that demonstrated a correlation between *AR* CAG repeats, TGCT histology, and disease progression, albeit the study size was limited [12, 13]. Grassetti *et al.* [10] observed that there was a larger variability of CAG than GGN repeats in both patients and controls, especially among those with rare alleles. When stratified, men with CAG repeats < 21 or > 24 were found to have a 50% and 76% higher risk of TC, respectively, than those with CAG 21–24. Therefore, the risk of developing TC seems to be lower for men with a CAG repeat number between 21 and 24.

In the meta-analysis, our first finding was that long GGN repeats were associated with an increased risk of TC, compared with repeats > 23; similarly, an increased risk was observed in studies with sample size > 200 and in the mid-latitude and seminoma subgroups. We speculated that GGN > 23 was associated with lower *AR* activity compared with the more common genotype with GGN ≤ 23, indicating that low androgen response could play a role in disease progression, which is consistent with the results of previous studies [10, 17].

Overall, the present meta-analysis reports for the first time the association between *AR* CAG and GGN repeat polymorphisms and the risk of TC. No significant association was observed between CAG repeat and TC in the models evaluated in the overall analysis, and the groups were heterogeneous. We then evaluated the source of heterogeneity in these groups. Furthermore, in the subgroup analysis of latitude, case size, control source, and histology, a significant association was found between CAG repeats and TC in the PB, mid-latitude, high-latitude, seminoma, and non-seminoma subgroups.

Interestingly, we observed no heterogeneity after stratifying according to latitude and histology. We found that CAG repeat polymorphisms with > 25 and < 21 + > 25 repeats were associated with an increased risk of TC in the mid-latitude subgroup but were associated with a decreased risk of TC in the high-latitude subgroup, indicating that latitude plays a key role in the effect of CAG polymorphism on the risk of TC. In addition, long CAG repeats reduced *AR* activity and increased the risk of TC in the mild mid-latitude environment. Previous studies have indicated that men with CAG repeats > 25 have lower androgen sensitivity [27, 28]. However, in the harsh and cold, high-altitude environment, long CAG repeats may protect against TC. This is because the exposure to different environments or lifestyle-related factors may have opposing effects on the male reproductive system [29].

In addition, we cannot exclude the possibility of the moderate effect of CAG repeat polymorphisms on the risk of TC due to marginal associations. These polymorphisms within or near the *AR* may drive malignant phenotypes. Therefore, large studies focusing on both gene-gene and gene-environment interactions are needed to explore the mechanism of testicular carcinogenesis.

However, this meta-analysis has some limitations. First, some studies with small sample size may not have enough statistical power to determine the real association and are thought to be more likely to report larger beneficial effects compared with larger trials [30]. Second, our results were only based on a Caucasian sample and polymerase chain reaction (PCR) sequences, and a more precise analysis would be conducted if more data were available. Third, clinical disorders are not the result of the disruption of a single gene, and genetic disruptions are embedded within the entire genome and are affected by environment exposure. In fact, other genes related to TC can also play a preeminent role in testis development.

In conclusion, we found that long GGN repeats were associated with an increased risk of TC compared with a reference group. Furthermore, an association between GGN repeats in *AR* and the risk of TC was found in studies with a sample size > 200 and in the mid-latitude and seminoma subgroups. We found that CAG repeat polymorphisms with > 25 and < 21 + > 25 repeats might confer a protective effect to the patients with TC in the PB, high-latitude, seminoma, and non-seminoma subgroups. However, it CAG repeat polymorphisms with > 25 and < 21 + > 25 repeats in the mid-latitude subgroup were associated with an increased risk of TC.

## MATERIALS AND METHODS

### Literature selection

Data from single reports were extracted (Figure 1). We searched PubMed and Web of Science until July 2015 to identify publications on the association between TC and CAG and/or GGN trinucleotide repeat lengths in *AR*. We focused on the studies performed in humans and on those that utilized the following key words: testicular cancer or TC, androgen receptor or AR, combined with CAG and/or GGN.

The inclusion criteria were as follows: (1) studies that evaluated the association between *AR* CAG or GGC/GGN repeat polymorphisms and the risk of TC; (2) studies with a case-control design; (3) studies that provided sufficient information on CAG or GGC/GGN repeat distributions between patients and controls; (4) studies for which the full text was available. Two reviewers assessed the full text of eligible studies from the above databases. Additional studies were identified by manually searching references of original and review articles on this topic.

### Data extraction and verification

Information on the enrolled studies is shown in Tables 1 and 2, including: (I) the first author's name; (II) year of publication; (III) country or region of origin, ethnicity, and method; (IV) number of cases and controls. Two authors (WJ Jiang and SM Liu) of the study extracted the information independently and screened the citations that met the inclusion criteria. The discrepancies were adjudicated via discussions until a consensus was reached.

### Data and statistical analysis

Data were divided into three categories: CAG repeat length of 21–25, which was used as the reference group, CAG length < 21, and CAG length > 25. The GGN repeat length was divided into 2 categories: GGN repeat length ≤ 23, which was used as the reference group, and GGN length > 23. To obtain specific data from these categories, we thoroughly analyzed and carefully obtained information from all the studies that met the inclusion criteria.

We predicted the contribution of *AR* CAG and GGN repeat polymorphisms to the risk of TC using the Stata software version 11.0. The OR with 95% (CI) was calculated to measure the strength of the associations [31]. A test of heterogeneity of the pooled results was performed using Cochran's *Q* test and Higgins I^2^ statistic [32]. *I*^2^ > 50% is considered as a measure of significant heterogeneity. If the result of the *Q* test was *P* > 0.10, the OR was analyzed using the fixed-effects model (Mantel–Haenszel method). Otherwise, a random-effects model (DerSimonian–Laird method) was used in cases of significant heterogeneity. Sensitivity analysis was performed to estimate the stability of the results by removing each study from the analysis, one at a time. Publication bias was evaluated using Begg's funnel plot. In addition to the visual inspection of the funnel plot, a value of *P* < 0.05 was considered to indicate the presence of significant publication bias [33–37].

## SUPPLEMENTARY MATERIALS FIGURES


