# Supplementary material for: Predictive value of GGN and CAG repeat polymorphisms of androgen receptors in testicular cancer: a meta-analysis
Source: Oncotarget. 2016 Feb 12;7(12):13754–64. doi: 10.18632/oncotarget.7337 (PMC4924676; doi:10.18632/oncotarget.7337)
Supplement: Supplementary file 1 [file oncotarget-07-13754-s001.pdf]

Predictive value of GGN and CAG repeat polymorphisms of androgen receptors in testicular cancer: a meta-analysis

Supplementary Materials

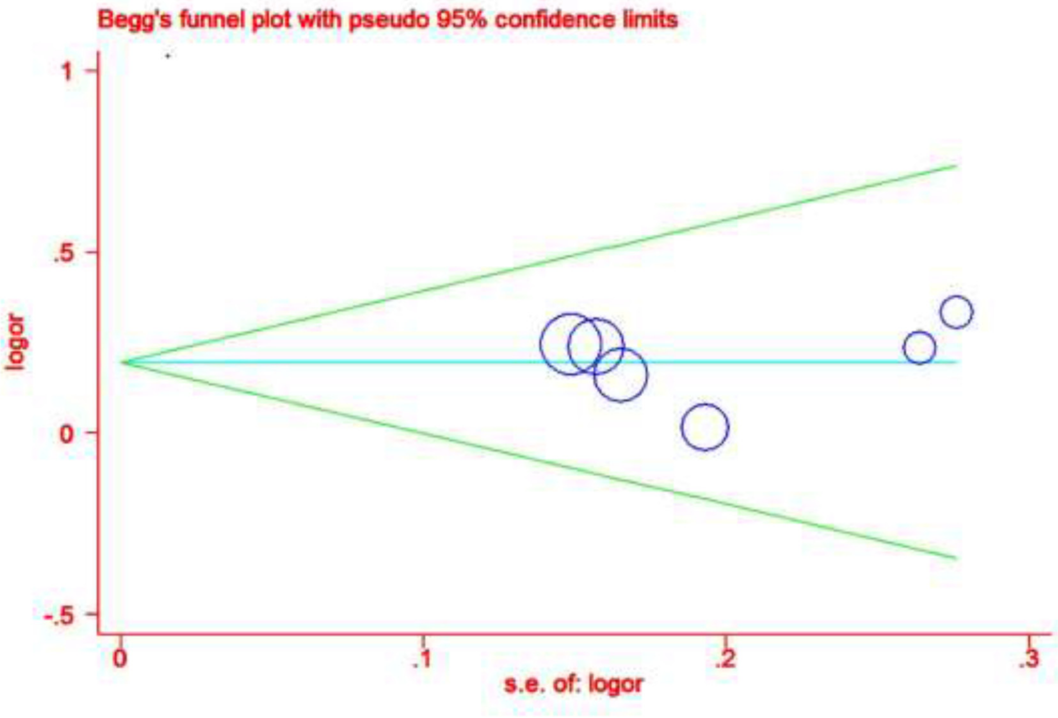

Supplementary Figure S1: Begg's funnel plot of publication bias test for GGN repeat polymorphisms. Each point represents a separate study for the indicated association. Log (OR), natural logarithm of OR. Horizontal line means effect size.

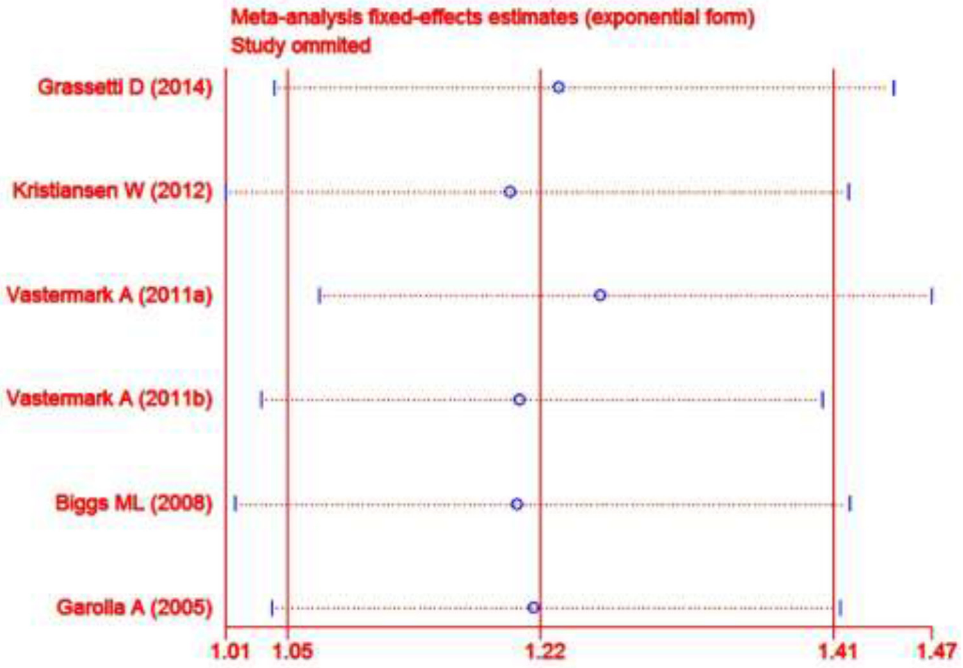

Supplementary Figure S2: Sensitivity analysis for GGN repeat polymorphisms. Studies are plotted according to the last name of the first author and followed by the publication year in parentheses. Each blue ring represents the OR point estimate. The ring (and broken line) represents the overall summary estimate, with confidence interval given by its width. The left, center and right unbroken vertical lines are successively at the lower CI limit, estimate and upper CI limit values.

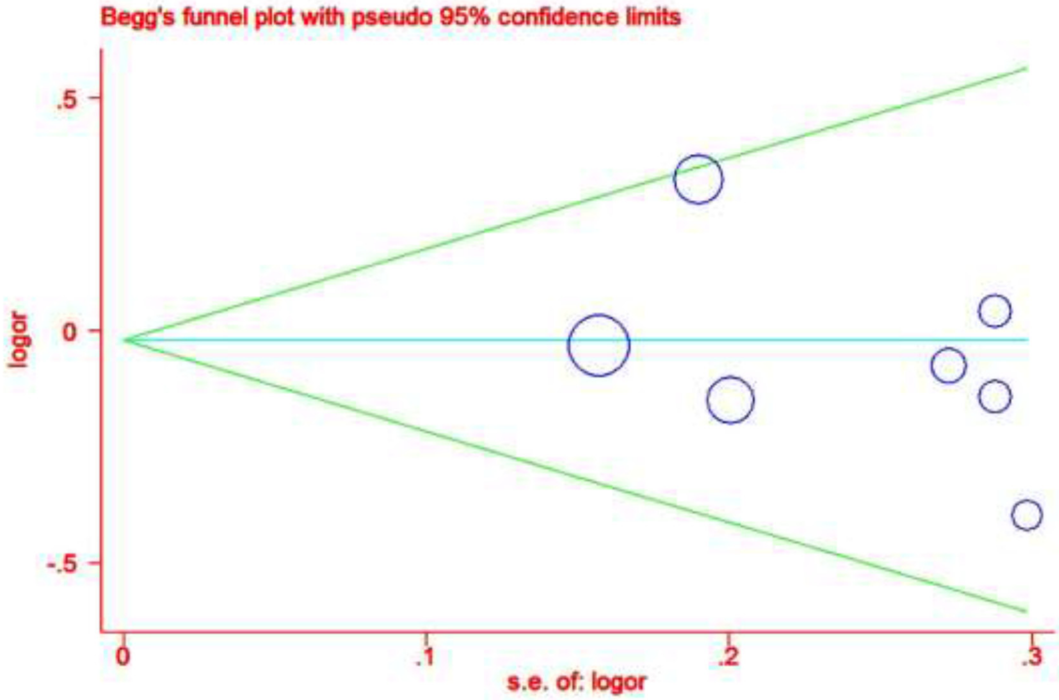

Supplementary Figure S3: Begg's funnel plot of publication bias test for CAG repeat polymorphisms under < 21 *vs.* 21–25 model. Each point represents a separate study for the indicated association. Log (OR), natural logarithm of OR. Horizontal line means effect size.

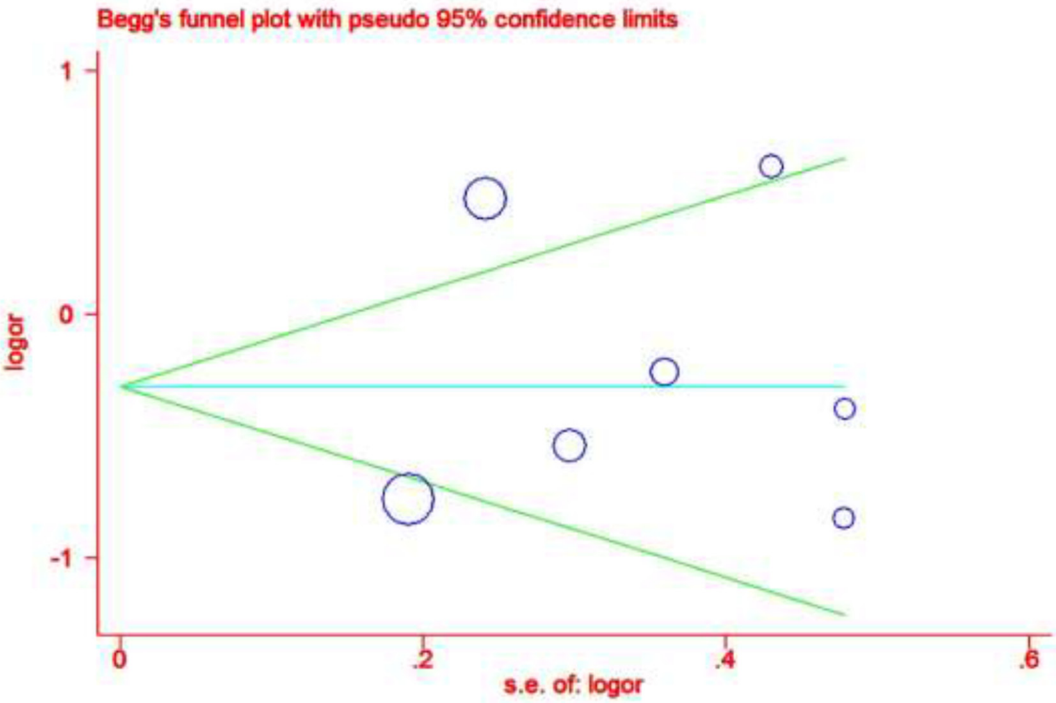

Supplementary Figure S4: Begg's funnel plot of publication bias test for CAG repeat polymorphisms under > 25 *vs.* 21–25 model. Each point represents a separate study for the indicated association. Log (OR), natural logarithm of OR. Horizontal line means effect size.

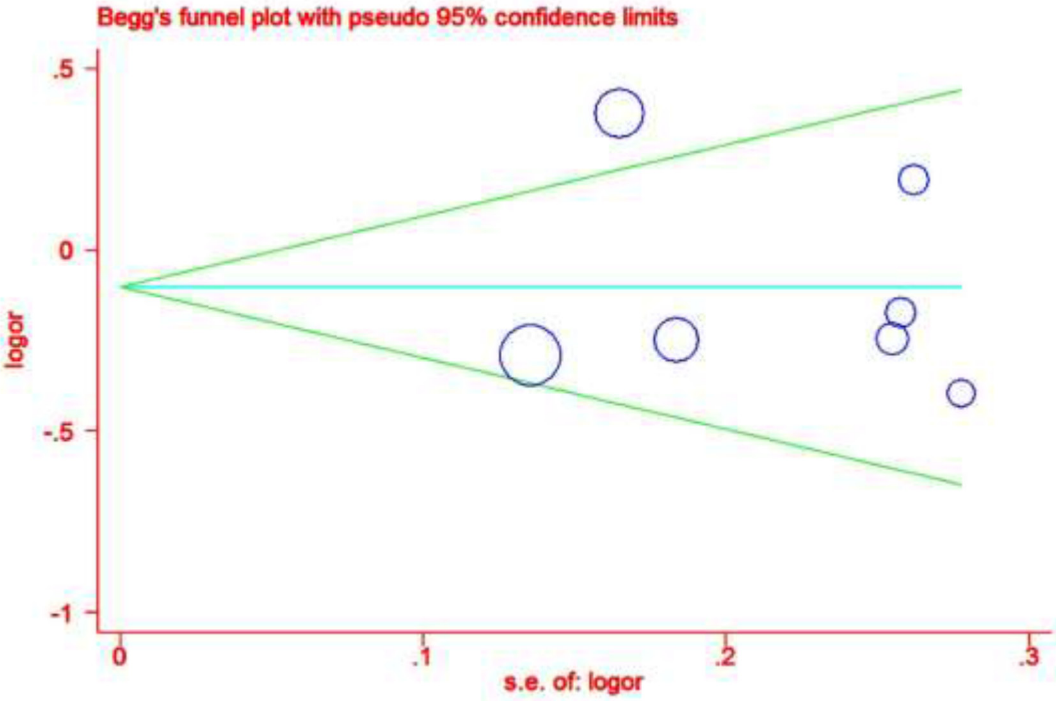

Supplementary Figure S5: Begg's funnel plot of publication bias test for CAG repeat polymorphisms under > 25 + < 21 *vs.* 21–25 model. Each point represents a separate study for the indicated association. Log (OR), natural logarithm of OR. Horizontal line means effect size.
